# Gaze Holding in Healthy Subjects

**DOI:** 10.1371/journal.pone.0061389

**Published:** 2013-04-26

**Authors:** Giovanni Bertolini, Alexander A. Tarnutzer, Itsaso Olasagasti, Elham Khojasteh, Konrad P. Weber, Christopher J. Bockisch, Dominik Straumann, Sarah Marti

**Affiliations:** 1 Department of Neurology, University Hospital Zurich, Zurich, Switzerland; 2 Departments of Otorhinolaryngology, Head and Neck Surgery, University Hospital Zurich, Zurich, Switzerland; 3 Department of Ophthalmology, University Hospital Zurich, Zurich, Switzerland; Barrow Neurological Institute, United States of America

## Abstract

Eccentric gaze in darkness evokes minor centripetal eye drifts in healthy subjects, as cerebellar control sufficiently compensates for the inherent deficiencies of the brainstem gaze-holding network. This behavior is commonly described using a leaky integrator model, which assumes that eye velocity grows linearly with gaze eccentricity. Results from previous studies in patients and healthy subjects suggest caution when this assumption is applied to eye eccentricities larger than 20 degrees. To obtain a detailed characterization of the centripetal gaze-evoked drift, we recorded horizontal eye position in 20 healthy subjects. With their head fixed, they were asked to fixate a flashing dot (50 ms every 2 s)that was quasi-stationary displacing(0.5 deg/s) between ±40 deg horizontally in otherwise complete darkness. Drift velocity was weak at all angles tested. Linearity was assessed by dividing the range of gaze eccentricity in four bins of 20 deg each, and comparing the slopes of a linear function fitted to the horizontal velocity in each bin. The slopes of single subjects for gaze eccentricities of ±0−20 deg were, in median,0.41 times the slopes obtained for gaze eccentricities of ±20−40 deg. By smoothing the individual subjects' eye velocity as a function of gaze eccentricity, we derived a population of position-velocity curves. We show that a tangent function provides a better fit to the mean of these curves when large eccentricities are considered. This implies that the quasi-linear behavior within the typical ocular motor range is the result of a tuning procedure, which is optimized in the most commonly used range of gaze. We hypothesize that the observed non-linearity at eccentric gaze results from a saturation of the input that each neuron in the integrating network receives from the others. As a consequence, gaze-holding performance declines more rapidly at large eccentricities.

## Introduction

Most healthy human subjects display a physiological centrifugal horizontal nystagmus at extreme lateral gaze in darkness ([1]). This ‘end-point nystagmus’ suggests that the gaze-holding system's performance noticeably degrades at larger eccentricities. The occurrence of end-point nystagmus is, however, quite variable and subjects showing no end-point nystagmus at all, regardless of eccentricity, have been reported ([2], [3], [4], [5]) while others show such nystagmus already at small gaze eccentricities ([2], [5]). These contrasting findings have been explained by the strong influence of the physical status of the subjects ([6] for review). For example alcohol consumption ([7], [8], [9]) as well as sleep deprivation ([5]) decrease the minimal horizontal gaze eccentricity at which end-point nystagmus appears.

In general, however, gaze shifts to moderate horizontal eccentricities evoke, even in darkness, only very weak centripetal eye drift in healthy subjects, as cerebellar control sufficiently compensates for the inherent leakiness of the brainstem gaze-holding network ([10], [11], [12], [13], [14]). Cerebellar disease unmasks the deficient behavior of the brainstem gaze holding system and leads to prominent gaze-dependent centripetal drift at small horizontal gaze-eccentricities, i.e. gaze-evoked nystagmus ([1], [15]). Patients affected by cerebellar disease often also show a transient nystagmus in the direction of the previous gaze eccentricity upon returning to primary gaze position after sustained eccentric fixation. This nystagmus, usually called rebound nystagmus ([16], [17]), is a consequence of a mechanism that reduces excessive drift velocity during a sustained eccentric fixation. Minimal rebound nystagmus has also been observed in some healthy subjects ([4], [18], [19]). Its presence in healthy subjects - although infrequent - suggests that physiological drift velocities may be sufficient to activate the adaptive mechanisms generating it.

To better understand the physiological and pathological manifestations of the inherent deficiencies of the gaze holding system, it is crucial to clarify how the centripetal horizontal eye drift grows in relation to eccentric gaze position. Several studies reported drift velocity for only one or very few specific horizontal gaze eccentricities (typically 30, 40 or 50 deg) ([3], [4], [18], [20]). This approach does not allow a detailed investigation of the relationship between the ability to hold gaze stable and concurrent eye eccentricity, but it is sufficient to characterize it under a simple modeling hypothesis: in order to obtain the eye position command for the ocular motor neurons, the velocity command needs to be integrated by a network of neurons, which is modeled as a leaky integrator ([11], [21]) with a time constant usually estimated between 10 s and 70 s ([1], [22]). Such a model results in an eye drift velocity that grows linearly with eye eccentricity with a slope equal to the reciprocal of the leaky integrator time constant ([11], [12], [21]).The cerebellum is hypothesized to provide a feedback loop in the model, which prolongs the time constant of the integrator, scaling down the slope of the eye drift with eye eccentricity ([23]). Early studies in patients ([24], [25]) observed nonlinear behavior in pathological nystagmus and therefore proposed modifications to the leaky integrator model, introducing an eye eccentricity dependent nonlinearity in the gain of the cerebellar feedback loop to account for nonlinear behavior. These nonlinearities were mainly considered to describe specific pathological conditions, although nonlinear growth of centripetal eye drift velocity with gaze eccentricity has also been observed in healthy human subjects ([3]). Nonlinear behavior at large eccentricities is not surprising since integration, the ability to maintain and update multiple levels of persistent activity, requires neuronal and network processes, that include nonlinearities such as the inhibitory cutoff in neuronal firing and possibly nonlinear synaptic transmission ([26], [27]). This supports a modeling hypothesis alternative to the leaky integrator, based on a network of neurons, whose properties mimic those observed from the neurons believed to be part of the gaze holding network. Such a model could explain both the leakiness and the nonlinearity, as they arise naturally from neuronal behavior. Additionally it could simulate the dependence of behavior on the tuning, which can be hypothesized to be under cerebellar control.

The purpose for the present study is, therefore, to characterize the relation between centripetal eye drift velocity and gaze eccentricity in healthy human subjects and clarify the limit of applicability of the single time constant leaky integrator model.

## Methods

### Subjects

Twenty healthy human subjects (8 females; mean age ±1 SD: 41±11 years; range 24–67 years) participated in the study. Informed consent of all participants was obtained in written form after full explanation of the experimental procedures. The protocol was approved by the Ethics Committee of the Canton of Zurich, Switzerland (Protocol N° E-33/2007), and was in accordance with the ethical standards laid down in the 1964 Declaration of Helsinki for research involving human subjects.

### Experimental setup

Participants were comfortably seated upright on a chair mounted on a two servo-controlled motor-driven axes turntable system (Tönnies D561, Freiburg i.Br., Germany; control system: Acutrol® ACT2000, Acutronic, Switzerland Ltd.). The two independent motor-driven axes are coincident and earth vertical. One rotates the chair and the other a cylinder (Optokinetik Drum, radius: 74 cm) mounted concentrically to the chair. Remotely controlled LEDs are attached to the cylinder at the level of subject's eyes. Safety belts around the feet and the shoulders restrained the subject. An adjustable chin rest and a forehead strap were used to stabilize the subject's head.

#### Recording of eye movements

Horizontal eye movements were recorded at 220 Hz with a head-mounted video-oculography (VOG) device (“EyeSeeCam”) ([28], [29]) consisting of swimming goggles with two mounted infrared cameras. A model of the eye rotation is used by the VOG system to derive the horizontal eye position from the pupil position recorded in the coordinate system of the cameras. An additional offline calibration was performed to improve the accuracy. Using the LED attached to the motorized cylinder, before the beginning of the experiment, we asked the subjects to look at a sequence of fixation points. We then fitted a second order polynomial function to the corresponding eye angles provided by the VOG system.

### Experimental procedure

Participants were asked to fixate a briefly flashing (50 ms every 2 s) red LED without moving the head. The LED was positioned at the level of the eyes in the range of horizontal gaze eccentricity from 40 deg left to 40 deg right. Each subject was tested in two subsequent runs, changing the order of presentation of the requested gaze eccentricities. Specifically the LED always started straight-ahead and slowly displaced (0.5 deg/s) up to 40 deg of eccentricity in one of the two possible directions (the initial direction was in one run leftward and in the other rightward, randomly selecting the first one), then the direction was reversed, continuing the displacement until the 40 deg of eccentricity of the opposite side was reached, when it was reversed again to return to straight ahead position, where it stopped. Both eyes were recorded simultaneously.

### Data analysis

Data analysis was done offline on a PC using interactive programs written in MATLAB (The Mathworks, Natick, MA), version 7.5. Velocity traces were obtained as the derivative of horizontal eye position traces. Saccades and blinks were interactively removed using a custom program that identifies all the data points exceeding by a given threshold the median velocity calculated over a time window moving in steps of one third of its width. The data points that exceeded the threshold at least two times were considered part of a saccade. The beginning and the end of each saccade were identified by searching for the closer reversals of the velocity. All data points belonging to saccades were removed. The threshold was set to 3 deg/s and the width of the window was 0.5 sec. Missing data or unreliable data (due to blinks, or if the pupil could not be reliably found at eccentric positions due to coverage by the eye lids) were not interpolated. We calculated median eye velocities recorded from every single subject within 4 non-overlapping, 20 deg wide bins of eye eccentricity (i.e. 0–20 deg and 20–40 deg for both sides), keeping the two runs (which differ by the starting direction), the two directions of target displacement and the two eyes, separated. Individual median eye velocities from all subjects were compared within each bin using a repeated measures three-way analysis of variance (Matlab function RMAOV33.m) ([30]) with the direction of target displacement, the run and the eye as factors, using post hoc Bonferroni correction to compensate for multiple comparisons.

The behavior of eye drift as a function of gaze eccentricity was analyzed using two separate procedures, one focused on single subject data and the other on pooled whole population data. The first provided a test of the reliability of the linear modeling, by testing the consistency of the parameters estimated by linear fit of different ranges of gaze eccentricity. The second allowed defining the general behavior of gaze holding, evaluating which function can best represent the growth of the drift velocity with gaze eccentricity.

### Subject-based data analysis

Instantaneous eye velocity values from both eyes, directions of target displacement and runs were pooled for each subject. The resulting data were sorted according to their eye eccentricity and then split in four 20 deg wide bins from 40 left to 40 right (i.e. 0–20 and20–40 for both sides). Under the assumption of linear behavior, the slopes obtained fitting the data from different bins should be the same within each subject. Calling *V* the instantaneous eye drift velocity and *E* the horizontal eye eccentricity the following function was fitted to each bin:



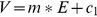
(1)


The linear slope *m* and the offset *c_1_* were optimized with an algorithm (Matlab function quantreg.m) ([31]) for quantiles regression minimizing a sum of squared residuals with respect to the median ([32]), as the assumption of normality of the data required for the ordinary least squares regression was not confirmed. Lilliefors test ([33]) indicated that the slopes in each bin across subjects were not normally distributed. To investigate the linearity of the behavior we performed a paired Wilcoxon signed rank test, between the coefficients estimated from the 0–20 deg bin and those obtained in the 20–40 deg bin, pairing those from each side. Additionally we calculated the ratio of the paired coefficients. A Wilcoxon signed rank test was used to test whether the ratios came from a population with a median different from 1. A median significantly smaller than 1indicates that the rate of growth of the drift velocity increased with gaze eccentricity, demonstrating that a linear fit does not capture the real behavior.

### Analysis of the pooled population

Each subject's instantaneous velocity was smoothed as a function of eye eccentricity using a weighted linear least-squares robust regression method (Matlab function smooth.m with “rloess” algorithm) based on a second order polynomial model ([34]) applied on a moving window equal to 20% of the whole range of gaze angles tested (16 deg). The results were interpolated every 0.1 deg from 40 deg left to 40 deg right. Lilliefors test ([33]) was consistent with the normality of the distribution of resulting velocities across subjects at each eccentricity. The mean velocity of all subjects was calculated at each interpolated eye position and fitted with Eq.1 and with the following equations:



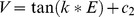
(2)

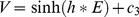
(3)where *tan* and *sinh* are the tangent and the hyperbolic sine functions, *k* and *h* are scaling coefficient and *c_2_* and *c_3_* are offsets. Variance accounted for ([35])was used to compare the quality of fit of the three functions. To check for possible distortions of the shape of the curve due to boundary effects of the smoothing process, we repeated the procedure decreasing the size of the moving window to 5% of the whole range of gaze angles tested (4 deg) and compared the obtained parameters.

### Mathematical model of a network simulating the gaze holding behavior

To illustrate how the underlying nonlinearities in the brainstem gaze holding networks could surface at extreme eye positions, we used a mathematical model of a network of neurons. The network simulates eye drift velocity by mimicking the physiological behavior of the neurons. The equations of the model were derived as follows.

Electrophysiological data have shown that, during eye fixations, neurons thought to be part of the neural integrator network in the brainstem ([36], [37], [38]) fire approximately linearly with eye position. Mathematically:



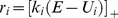
(4)


The subscript indexes the neurons; *r* is the firing rate and *E* the eye position. The two parameters in this expression are the slope, *k_i_*, and the eye position threshold, *U_i_*, which can be measured experimentally. The inhibitory cutoff (there are no negative firing rates) is the only nonlinearity considered in equation 4. We simulated a bilateral network of rate neurons based on published work ([26], [39]) composed of 40 neurons (20 per side). In order to simplify, we consider that the same neurons can lead to both excitatory and inhibitory postsynaptic currents. The firing rate of the neurons in the network is determined by the total amount of input current: excitatory contributions from ipsilateral neurons, inhibitory contributions from contralateral neurons, a tonic input *T* and a command input *B*. The postsynaptic current is characterized by a synaptic activation variable *s*, which determines the active proportion of the maximum synaptic current. We write the weight of the connection from neuron ‘j’ to neuron ‘i’ as a product of two factors: the postsynaptic factor 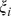 and the presynaptic factor 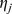. NMDA synaptic transmission, with decay time constant on the order of 100 ms, is hypothesized to play an important role in network based persistent activity ([40]). Therefore we will be working in a regime in which synaptic dynamics is slower than firing rate dynamics and we can consider that the firing rate adapts instantaneously to the synaptic input dynamics. In such cases, for a right side neuron *i*, we can write ([41]):



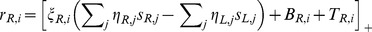
(5)


A similar relation can be written for neurons in the left side population. To model the synaptic current that each neuron creates in the postsynaptic neuron, we use a synaptic activation function 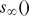 with first order dynamics ([26]):



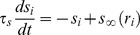
(6)with 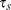 = *100 ms*, as suggested by Seung and colleagues ([26]). As mentioned above, this is the order of magnitude of the decay time constant of postsynaptic currents through NMDA receptors. It follows from the above equation that all firing rates depend on the combination:



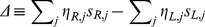
(7)whose dynamic equation can be obtained by differentiating equation 5 and using equations 5 and 6.



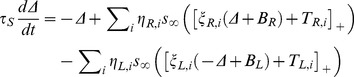
(8)


That is, the dynamics of the network can be reduced to a single equation. This network will maintain stable fixations for any values of Δ for which the right hand side of equation 5, itself a function of Δ, is zero in the absence of driving inputs (when *B_R_* and *B_L_* are zero). For such stable values, equation 5 gives:



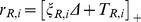
(9)


Comparing with equation 4, it is possible to assign Δ to the internal representation of eye position, *ξ* to the slope of the tuning function and *T* to the combination –*kU*. The values for *ξ* and *T* can be obtained from neurons' tuning curves, which are experimentally accessible ([39]). We generated these values by choosing equally spaced eye position thresholds within right and left populations of the network and by assigning slopes that are slightly increasing as eye thresholds become more ipsilateral to anatomic location of the neurons. The presynaptic factors *η* are undetermined and can be used to minimize the right hand side of equation 8 in the absence of eye movement commands. We considered both linear and saturating forms of the activation functions *s_∞_* and used equation 8 to find the values of *η* that minimize the drift in Δ, which is expressed by the cost function in Eq.10.



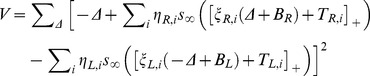
(10)


In the dark, the ability to maintain fixations depends on the behavior of equation 8, which gives the drift as a function of Δ. In our simulations, we will consider Δ as a proxy for eye position and derive position velocity plots (PV plots) directly from equation 8.

## Results

**Figure 1:**
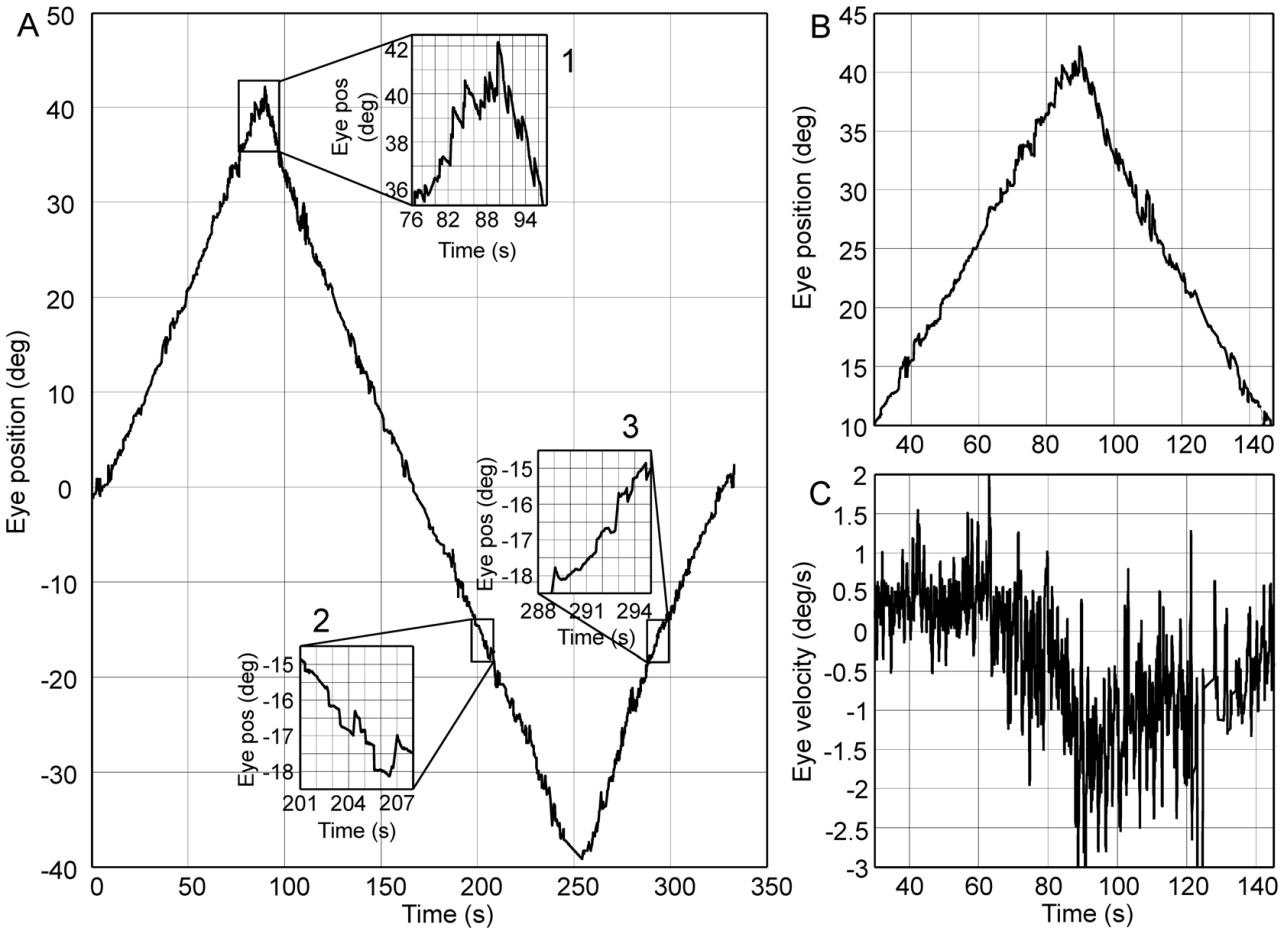
Raw data recorded in a single trial from a typical subject. Panel A - Left eye position plotted as function of time. Positive angles correspond to right eccentricities as seen by the subject. In this trial the dot was moving (0.5 deg/s) rightward at first. Inset 1: At extreme eccentricities the centrifugal beating nystagmus is clearly visible and the slow phase shows the tendency of the eye to return toward the primary position. Inset 2 and 3: Difference in the slope of the position trace at the same eccentricities when the dot is moving outward or inward. Panel B and C - Position (panel B) and velocity (panel C) of the eye at eccentricities larger than 10 deg right. The eye velocity begins to decrease from its baseline before the onset of the nystagmus, showing the growing centrifugal drift. Note that the baseline velocity is not zero but is positive between 10 and 25 deg of gaze eccentricity. When returning to 10 deg, however, the velocity is negative, showing the asymmetry in the baseline velocity showing the subject's attempt to match the target displacement velocity.

Figure 1 shows left eye position recorded in one typical subject as a function of time. The presence of a centripetal drift is evident at extreme gaze eccentricities, where a clear end-point nystagmus appears (inset 1 in figure 1-panel A). It is, however, noticeable (inset 2 and 3 in figure 1-panel A) that for lower eye eccentricity the eye position trace did not show centripetal drift. On the contrary, the eyes position displays a rather constant slope, which appears to be related to the direction of target displacement. This is confirmed when looking at the velocity trace in panel C of figure 1, representing the velocity corresponding to eye eccentricities larger than 10 deg (figure 1 - panel B). The eye velocities recorded at 10 degree of eccentricity with the target moving in opposite directions (the two ends of the plot in panel C) have two different values, one for each direction of target displacement, which nearly match the rate of the target displacement (±0.5 deg/s). Panel C in figure 1 also shows that the effect of the deficiency of the gaze holding system is already visible at 25 deg (around 60 s, according to figure 1- panel B), where the end-point nystagmus is absent, but the positive velocity value observed at 10 deg of eccentricity began to decrease.

**Figure 2:**
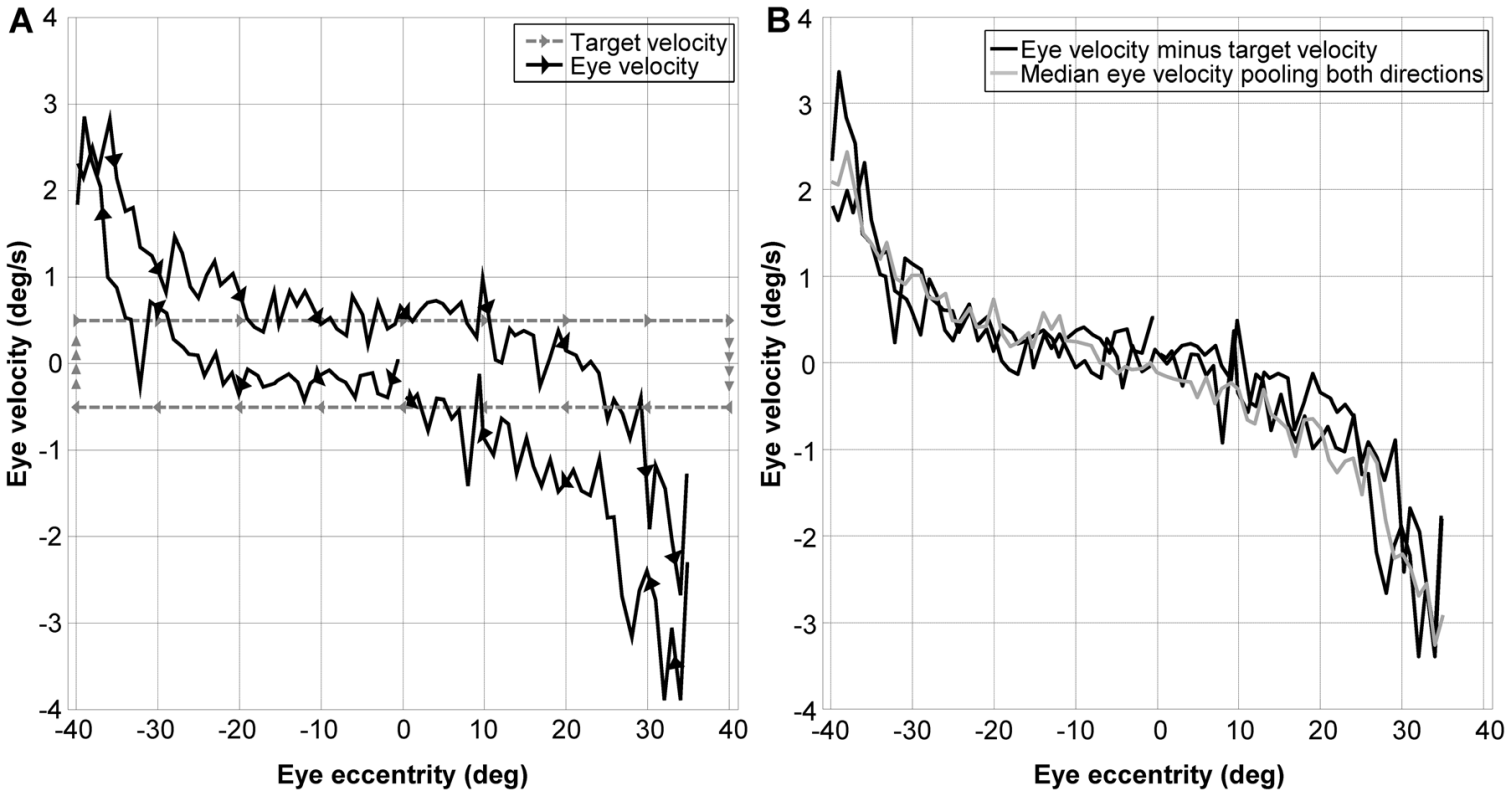
Position-Velocity plot considering target direction. Panel A - Black lines: Medians of the eye velocity within a 1 deg-wide bin plotted as a function of gaze eccentricity keeping the direction of target displacement separated. Gray lines: velocity of the target as a function of its position during the whole recording period. Note that the eye velocity matches the target velocity from the beginning suggesting that the offset observed around the straight ahead gaze is not due to a memory effect. The arrows show the directions of target and eye displacement. Panel B - Black lines: Velocity traces from the left panel after subtracting the correspondent target velocity. Gray line: Medians of the eye velocity within a 1 deg-wide bin plotted as a function of gaze eccentricity after pooling data from different directions of target displacement.

The constant value of the velocity signal observed in figure 1 (panel C – left end) between 10 deg and 25 deg of eccentricity suggests the presence of a velocity signal, that, at higher eccentricity, is summed to the position dependent centripetal drift that represents the object of our study. To understand how to best account for such a signal when estimating the eye centripetal drift, we plot the medians of the eye velocity recorded within single bins (width = 1 deg) centered at every degree of eye eccentricity in the range tested as a function of gaze, keeping data obtained with the target moving in opposite directions separated (figure 2). As already observable in panel C of figure 1, an eye velocity matching the one of the target displacement is clearly visible at low eccentricity. This is also confirmed by the right panel of figure 2, where the difference between the eye velocities recorded with target moving in opposite directions is reduced after subtraction of the target velocity.

To find out whether the direction of target displacements or other factors affect the recorded instantaneous eye velocity, we calculated for each subject the medians of the velocity within four non-overlapping 20 deg wide bins in the range tested, keeping the different runs (which differ by the starting direction), the different directions of the target displacement and the two eyes separated. Pooling the data of all our subjects, we used a repeated measure three-way ANOVA within each bin separately. After applying Bonferroni correction for multiple comparisons, we found that the run (i.e. initial displacement toward right vs. initial displacement toward left) and the eye (i.e. left vs. right eye) were not associated with a significant difference in any bin. The direction of target displacement was instead a significant factor (p<0.001; F(1,38) = 32.5 and F(1,38) = 39.8 for left and right eccentricities, respectively) in the two central bins (between 0 and 20 deg on both sides), with higher horizontal eye velocities when the target was moving toward the subjects' straight-ahead position, but not in the two outer bins (between 20 and 40 deg on both sides). The median difference between eye velocities recorded at the same eccentricity with the target moving in opposite directions was 0.45 deg/s; approximately half of the value expected considering the two opposite velocity offsets needed to match the target displacement in both directions. This finding, together with the statistical analysis, suggests that the velocity signal observed in figure 1 varied mainly with eye eccentricity and cannot be subtracted as a direction dependent offset. Therefore we pooled the data from both directions at a given gaze angle to cancel the velocity signal, which causes the difference between the two directions of target displacement (figure 2). The resulting median velocity represents the eye centripetal drift velocity due to the gaze holding deficiency.

To investigate the behavior of eye drift as a function of gaze eccentricity we applied two separate analyses to our data: one fitting single subject data and the other evaluating the average of the whole population.

**Figure 3:**
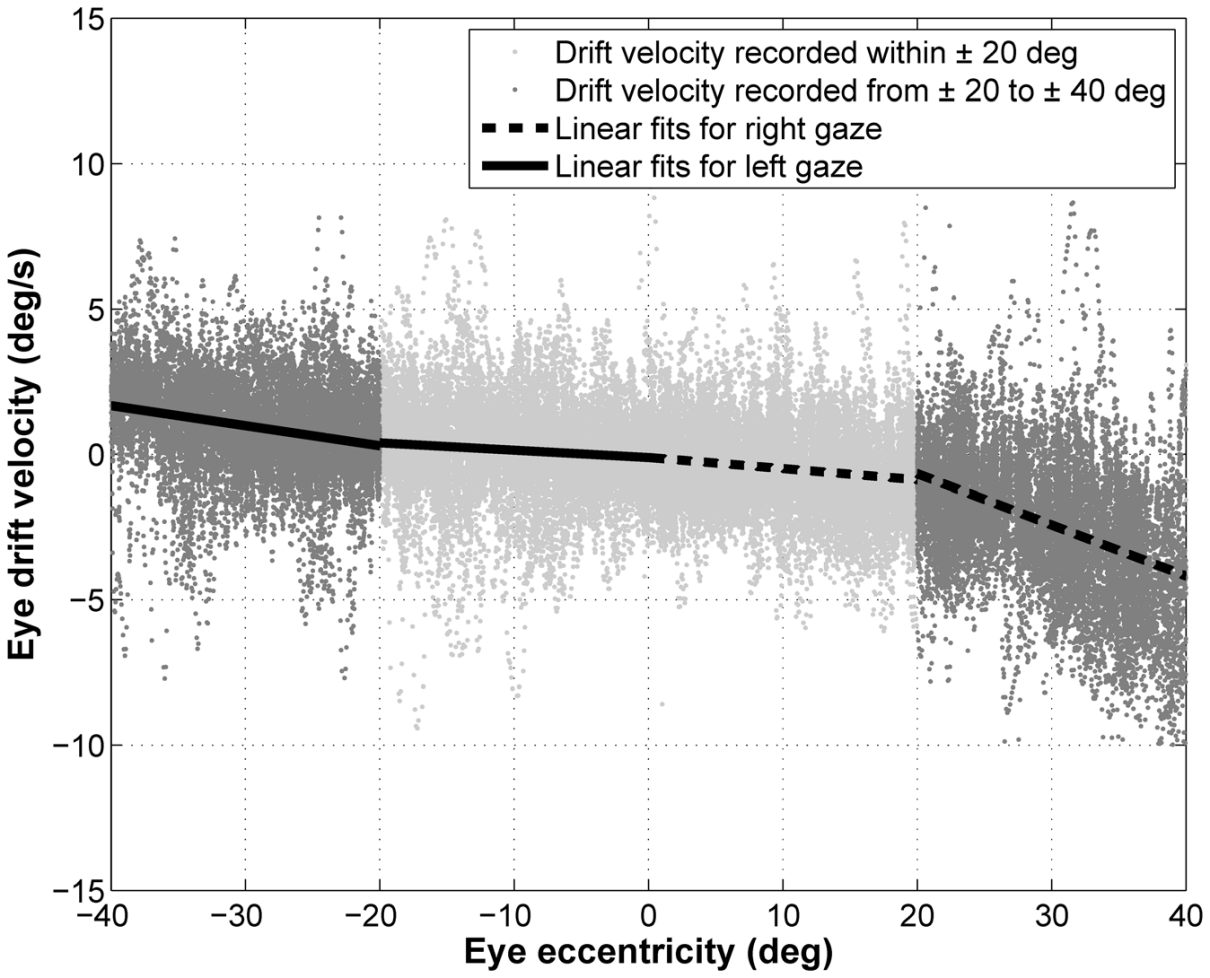
Position-Velocity plot and linear fit. Gray dots: Instantaneous velocity plotted as a function of the eye eccentricity. Light gray dots: Velocity in the 0–20 deg bins; dark gray dots: Velocity in the 20–40 deg bins; black line: linear fit of the velocity in the 0–20 deg bins and in the 20–40 deg bins.

In the first of these analyses, instantaneous velocity recorded from each single subject was sorted as a function of gaze eccentricity, pooling data from the two eyes, the two runs and the directions of target displacement. Data of each subject were separated in four bins defined as for the statistical analysis above. We fitted a linear function of the eye eccentricity (Eq.1) to the values in each bin separately. Figure 3 shows an example of this fitting procedure in a typical subject. The goal of this analysis was to quantify the reliability of the parameters estimated by a linear fit for different ranges of gaze eccentricity.

Using a paired Wilcoxon signed rank test we found that the slopes fitted from each subject for gaze eccentricities between 0 and 20 on one side were significantly (p<0.05) smaller than those obtained in the same subject for gaze eccentricities between 20 and 40 deg on the same side. The median ratios (median absolute deviation in square brackets) of the paired slopes were 0.44 [0.29] on the left side and 0.32 [0.25] on the right side, respectively. Ratios of the slopes were significantly lower than 1 (p<0.01), confirming the significant increase of the rate of growing of the eye drift velocity with gaze eccentricity and therefore indicating a non-linear behavior. Pooling both sides the median ratio was 0.41 [0.29]. The mean slopes of the fitted subjects are reported in table 1.

**Table 1:** Summary of the drift velocity and the slopes estimated from single subjects.

	0 deg to 20 deg	20 deg to 40 deg
**Left gaze**		
Median	0.33 ± 0.17	1.21 ± 0.56
Slope	−0.021±0.014	−0.045±0.022
Ratio to 20–40 deg	0.44 [0.29]	1
**Right gaze**		
Median	−0.36 [0.30]	−1.31 [0.54]
Slope	−0.020[0.010]	−0.047[0.045]
Ratio to 20–40 deg	0.32 [0.25]	1

**Figure 4:**
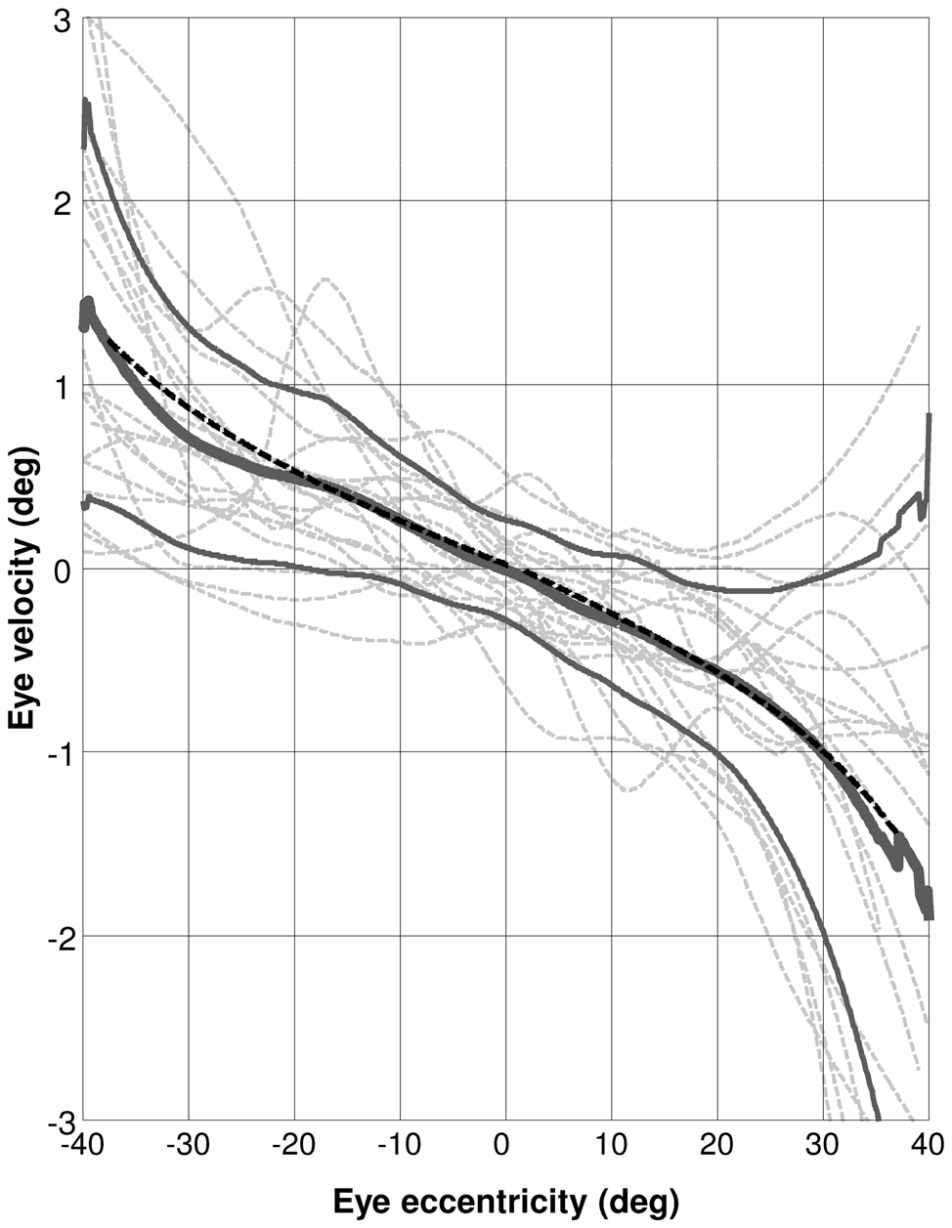
Smoothed Position -Velocity plot of the whole population. Dashed gray lines: Individual position-velocity curves obtained after smoothing and interpolating instantaneous velocity as a function of eye eccentricity; solid thick gray line: mean of the smoothed individual position-velocity curves; solid medium gray line: mean ±1 standard deviation of the smoothed individual position-velocity curves; dashed black line: tangent fit of the mean of the smoothed individual position-velocity curves.

The second analysis aimed at identifying a function that better represents the drift behavior, showing an improvement over the linear one. We characterized the behavior of the whole population by smoothing the individual instantaneous eye velocity traces of all subjects as a function of gaze eccentricity and interpolating them for all angles between 40 deg left to 40deg right in steps of 0.1 deg. Lilliefors test ([33]) confirmed that the obtained eye velocities at every step of interpolated eccentricity can be assumed to be normally distributed across subjects. We therefore calculated the mean eye velocity at each interpolated gaze eccentricity (figure 4) and fit each side with a linear, a tangent and a hyperbolic sine function (Eq. 1, Eq.2 and Eq.3, respectively). The variance-accounted-for evaluated in the range 0–20 deg scores were 0.90 and 0.97 for the linear fit on the right and left side, 0.98 and 0.95 for the tangent fit and 0.95 and 0.96 for the hyperbolic sine fit on the right and left side, respectively. When evaluating the fit in the range 20–40 deg these values however dropped considerably for the linear fit (0.84 and 0.81 for the right and the left side respectively) and moderately for the hyperbolic sine fit (0.92 and 0.87) but were almost unaffected for the tangent fit (0.98 and 0.92). The slopes of the linear fit were −0.036 s^−1^ on the right and −0.029 s^−1^ on the left side, corresponding to a time constant of 28 and 34 s, respectively. The values of *k*, the scaling factor of the tangent function, that provide the best fit of the data were 1.53 and 1.28 for right and left side, respectively. The values of *h*, the scaling factor of the hyperbolic sine function, that provide the best fit of the data were 0.031 and 0.025 for right and left side, respectively. To check for possible distortions due to boundary effects of the smoothing procedure, we repeated the analysis reducing the width of the moving windows used to smooth from 16 deg to 4 deg. The estimated parameters changed by less than 2% of their previous values, proving the robustness of our estimate to boundary effects.

### Results of the simulations of a network of neurons

**Figure 5:**
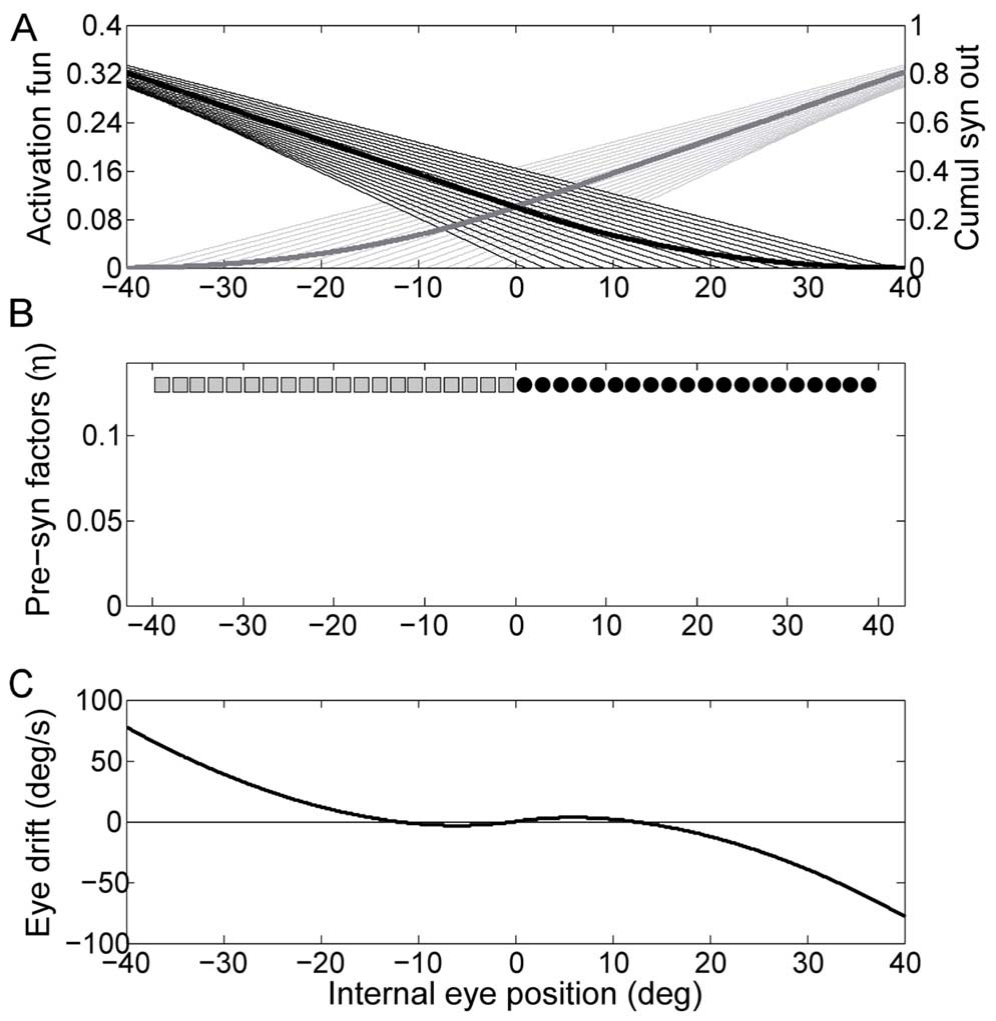
Simulation of the network without tuning. Panel A shows the output of the synaptic activation function of each neuron (thin lines) as a function of the internal representation of eye eccentricity (Δ), the zero of each line indicates the neuron threshold, i.e. the eccentricity at which the inhibitory cutoff takes place. The thick lines are the cumulative output of both sides of the network, obtained by combining all the synaptic activation functions with their factor *η*. Panel B shows the presynaptic factor *η* of each neuron, indexed according to the threshold shown in the upper panel, here set to the same value for all neurons to illustrate the general features of a non-tuned network. Panel C shows the PV plot for the internal representation of eye position.

To show how the observed behavior can stem from the nonlinearities that affect the integrator network at the neuronal level, we simulated a mathematical model of a network incorporating some of the known characteristics of those involved in the velocity signal integration in the goldfish and showing the effect of different tuning of the free parameters (see Methods). Without tuning (using the same value for every *η*), the drift grows rapidly with eye eccentricity (figure 5). Interestingly, however, the overall shape shows a clear nonlinear behavior evidencing the intrinsic nonlinearity of the drift pattern due to the influence of the neurons' natural nonlinearities (the only nonlinearity considered in this simulation is the inhibitory cutoff since we assumed a linear synaptic activation function).

**Figure 6:**
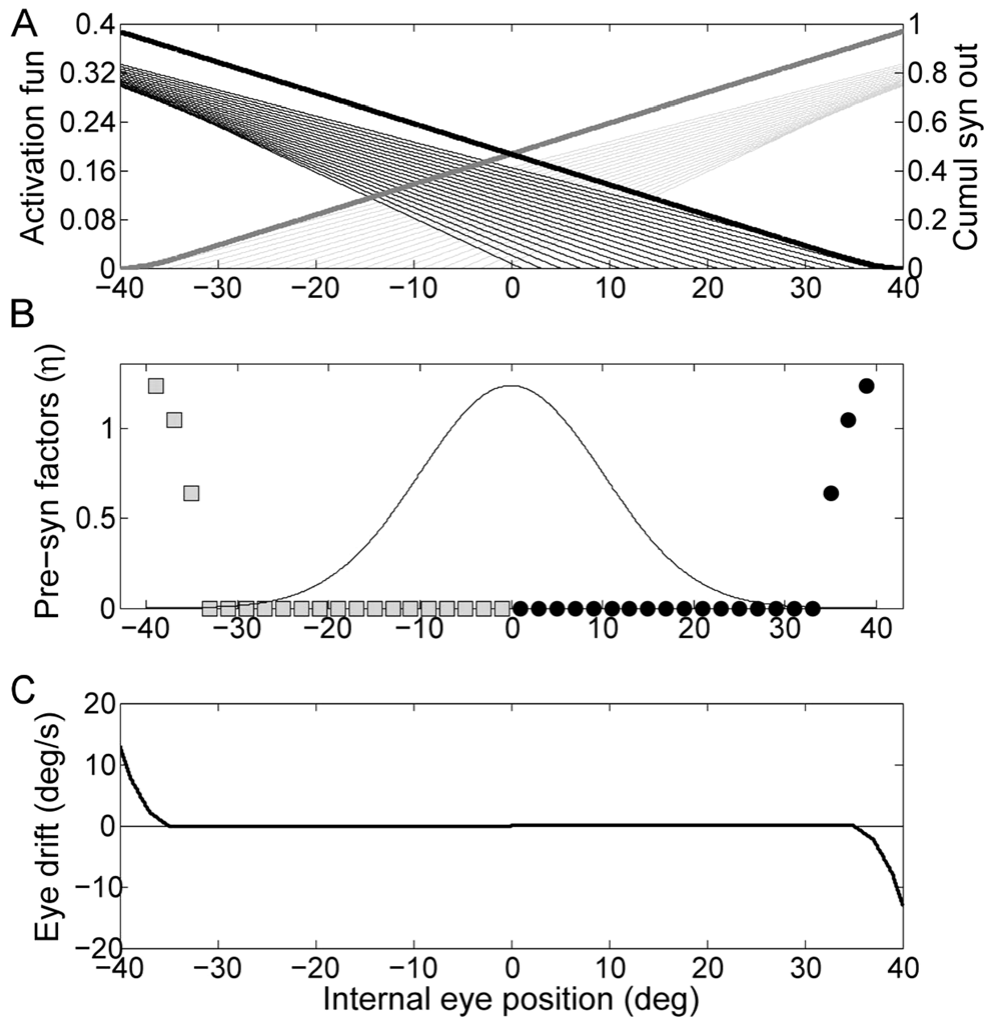
Simulation of the network tuned with non-uniform error function. The Gaussian function of the eye eccentricity represented by the solid line in the central panel has been multiplied to the resulting drift, i.e. the error to minimize, during the optimization procedure. The contents of the panels are as in figure 1A.

As the eye spends less time in the most eccentric positions than in the center, we considered a reasonable assumption to use a non-uniform tuning procedure, which will weigh more the drifts occurring in more central eye positions. This can be simulated by weighting Eq.10, which represents the error to minimize, with a Gaussian function of the eye eccentricity Δ (figure 6). We obtained almost perfect integration in the central eye range, corresponding to the almost flat region in figure 6 (bottom panel). Outside this range the performance of the network decreases dramatically and the drift explodes.

**Figure 7:**
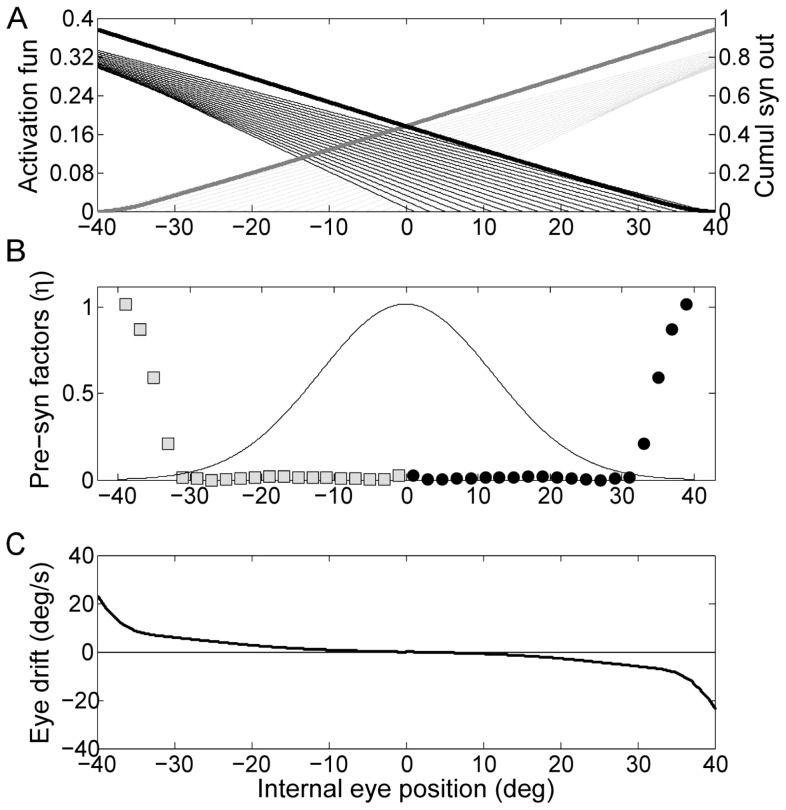
Simulation of the network tuned favoring leakiness against instability. The inverse of the Gaussian function of the eye eccentricity shown by the solid line in the central panel has been used as a gaze-dependent threshold for the non-penalized drift during the optimization procedure. The contents of the panels are as in figure 1A.

If we set a gaze-dependent tolerance for allowed drift in the minimization procedure, simulating a mechanism favoring leakiness over instability, it is possible to obtain a plot (figure 7) qualitatively resembling the mean trace depicted in figure 4. This was obtained by zeroing all the values of Eq.10 below the inverse of a Gaussian function of the eye eccentricity Δ and which will cause a centripetal drift.

**Figure 8:**
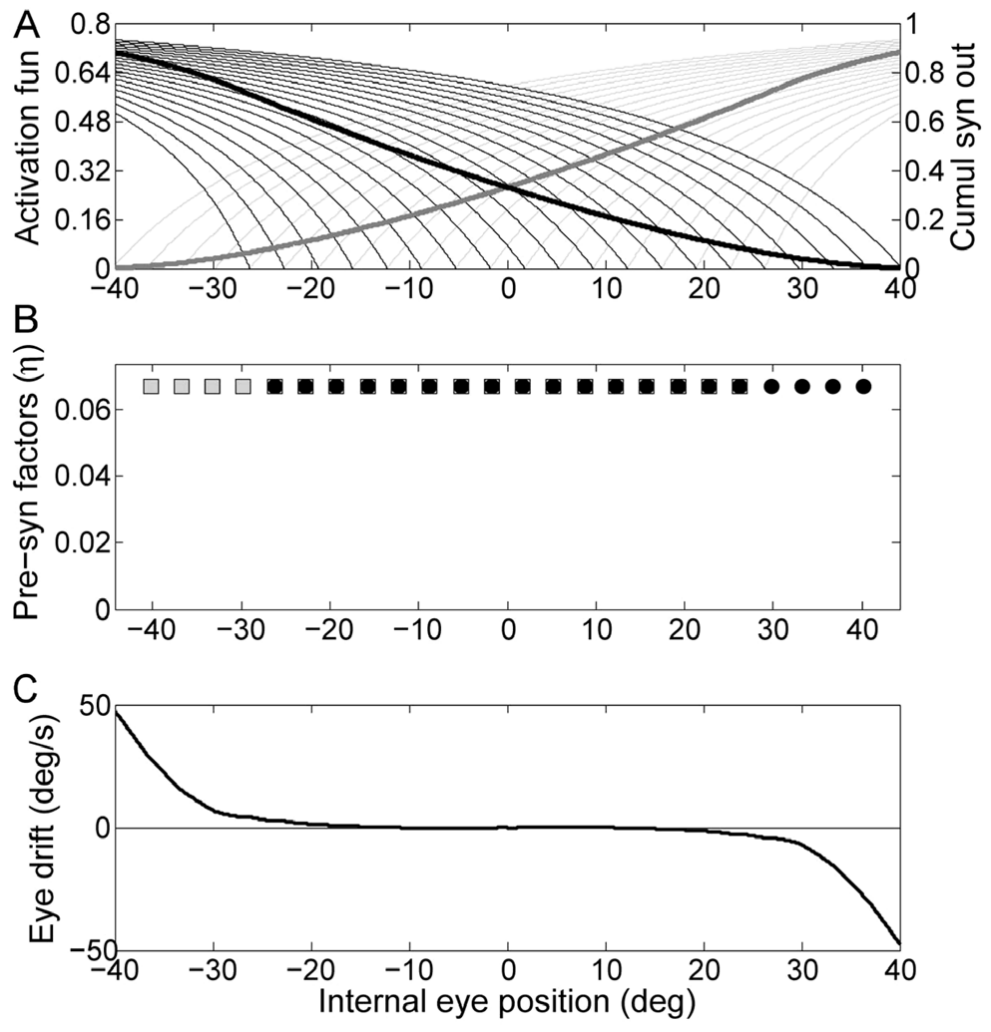
Simulation of the network using nonlinear synaptic activation functions. The contents of the panels are as in figure 5.

To illustrate that different hypotheses in the network design can also generate simulations that mimic the experimental data, we include a simulation with saturating synaptic activation functions and a partial overlap of the activation thresholds in the center of the eye position range (Figure 8, constant weights).

**Figure 9:**
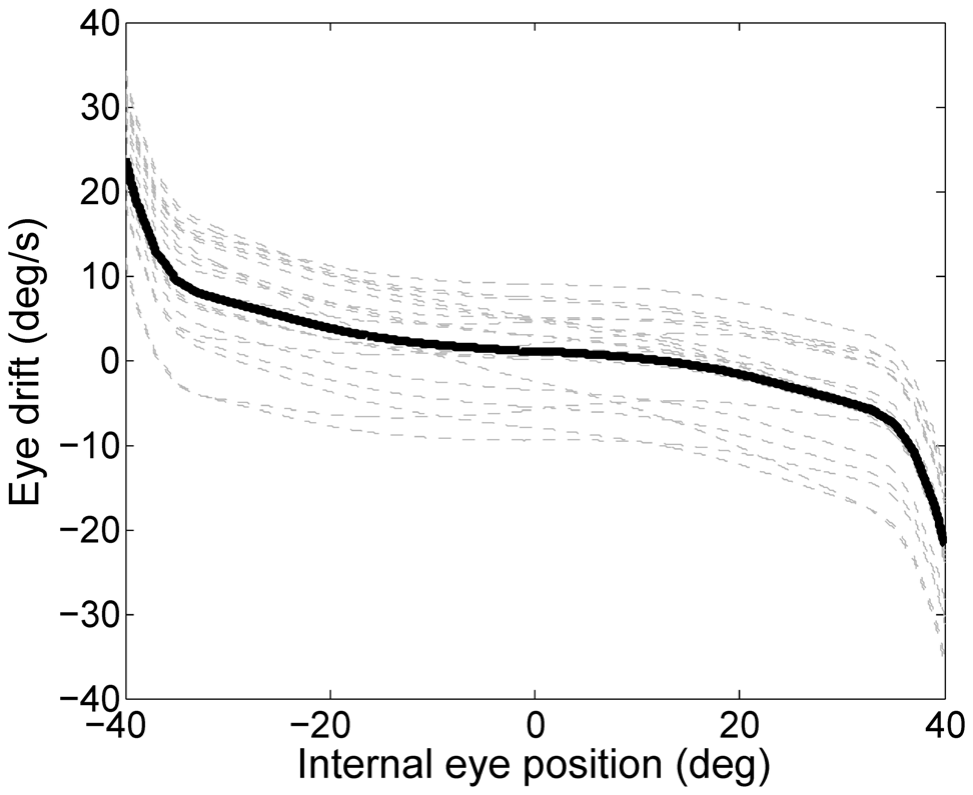
PV plots of random perturbations of tuned network. Effect of modifying the tuned values of *η* by a random fraction of 5% of the value used in figure 7. Dashed gray lines shows 20 different perturbations. The black solid line represents their mean.

Random perturbations around the weights used in Figure 7, generated patterns similar to Figure 4 in the main text (figure 9).

## **Discussion**

Centripetal drift of the eye in eccentric positions is a known phenomenon possibly caused by non-ideal integration of the eye velocity command when generating the position command for the motoneurons driving the eyes ([1] for references). This process is usually approximated by a leaky integrator ([11], [21]) with a time constant that ranges between 10 s and 70 s ([22]). Such approximation implies that the drift velocity grows linearly with gaze eccentricity, with a slope equal to the inverse of the time constant. Although it has been shown in a few studies that this approximation may not hold for all eccentricities ([2], [3]), a detailed characterization of the gaze dependent centripetal drift was missing.

In this study we investigated gaze-holding performance in healthy human subjects by measuring eye drift velocity as a function of gaze eccentricity over a ±40 deg range. Pooling all subjects, we found a clear drift pattern with approximately linear behavior only within the central 20 deg of gaze eccentricity. For larger eccentricities the slope increased gradually, resulting in a curve that was better fit by a tangent function (figure 4). Our results therefore contradict the assumption of linearity of horizontal drift velocity with respect to eye eccentricity, showing that modeling the gaze holding network as a leaky integrator with a single time constant ([11], [21]) might be misleading if used on eye eccentricities larger than 20 deg. According to our data, this assumption is indeed consistent with the observed behavior only in a limited range around the primary position, where the linear fit scored the same variance-accounted-for value as the tangent one. Such a range is likely to coincide with the most commonly used eye position, since sustained horizontal gaze exciding 30 degrees is quite rare under normal conditions as head rotations integrate gaze shifts when exploring visual scene. This may suggest that the gaze holding system is optimized to behave linearly within a given range, while at larger eccentricities it gets nonlinear, an observation potentially relevant when investigating pathological forms of nystagmus, like gaze-evoked nystagmus and rebound nystagmus.

It may be argued that the specific characteristics of our paradigm affected the recorded drift velocity. In contrast to the reported earlier studies that used large gaze shifts between different recorded positions, we slowly displaced the target to obtain a sequence of quasi-continuous position steps. This allowed us to minimize the distance between the evaluated gaze eccentricities, keeping the recording time short, and not sacrificing the range tested. A saccade-based paradigm usually requires the subject to rapidly look eccentrically to elicit centripetal drift, and to look back to straight head after each trial to guarantee the same starting condition in each trial. This approach is inefficient if one aims at acquiring the same number of eccentric gaze positions as we recorded, as it would require two gaze shifts for every gaze eccentricity and only the very first second of every eccentric fixation could be used. For our experimental setup, which uses LEDs embedded in a motorized drum surrounding the subject, a quasi-static displacing target was a good compromise between the efficient data acquisition and recording time. We reasoned that, considering the characteristics of the system, there is, in principle, no reason to prefer one method over the other, as both require the integration of a velocity command to reach the desired eccentricity and none of the two guarantees that the possible nonlinearity of the integration network will not affect the estimation of the centripetal drift. For small gaze angles we found an evident velocity offset of a magnitude similar to the velocity of target displacement. This offset caused a significant difference between the instantaneous velocities recorded when the eye moved rightward or leftward (figure 2). The strong similarity of the left panel of figure 2 with a hysteresis trace may suggest the hypothesis that a memory-like effect, developed when reaching large gaze eccentricities, is responsible for the offset. However, since the offset is immediately present during the first outward directed movement (see figure 1 and 2), it cannot be caused by a hysteresis phenomenon. We hypothesized that such an offset results from the velocity command needed to keep the eye on the flashing target, estimated by the brain by extrapolation from the displacement of the flashing target observed over time, possibly through the smooth pursuit system. Since the difference between rightward and leftward directed movements disappeared with larger eccentricities, subtraction of target velocity was not justified. A possible explanation is that the smooth pursuit gain decreased with eccentricity or that the integration of the velocity command becomes less efficient the more the eyes moved away from the center, causing a decrease of the observed velocity offset. Although theoretically it can be argued that the offset can bias the analysis of the centripetal drift velocity, we assumed that, by pooling the velocities recorded with the target moving in both directions, the effect of the two offsets into opposite directions would cancel out. A perfect cancellation would be obtained, if the smooth pursuit gain was symmetric in the two directions, as the offset would have the same magnitude at each given eccentricity but opposite signs when the target moved in opposite directions. In the case of an asymmetry between leftward and rightward smooth pursuit gain, the effect on our data would be that of adding a vertical bias to the whole velocity curve, causing a non-zero velocity when the subject is looking straight ahead, which is not present in our data and that would not affect the nonlinearity of the behavior in any case.

Although the nonlinear behavior emerges clearly when considering the population mean curve, the high variability across subjects, shown by the light gray dashed lines in figure 4, representing smoothed position-velocity curves of single subjects, make more difficult to observe a clear behavior when considering single subject data. Nonetheless, we found that, within subjects, the slopes of a linear function changed significantly (p<0.05) when fitting different portions of the range of recorded gaze positions. Specifically, between 0 deg and 20 deg of eye eccentricity the slope of the drift velocity was, in median, 0.41 times the one obtained between 20 and 40 deg. Such a ratio, which was significantly (p<0.01) lower than 1, confirms the nonlinear behavior evidenced by the population approach discussed above.

Considerable variability between subjects is a common observation in both the papers on end-point nystagmus reporting eye drift velocities ([3], [18]) and in those discussing the angle of nystagmus onset ([2], [4], [5], [42]). This is usually explained by the strong influence of the physical status of the subjects ([6] for review). A direct comparison with results from previous studies ([3], [18]) must be made with caution because of differences in the range of gaze eccentricities tested. Gordon and colleagues ([18]) reported an eye drift velocity of 0.3 deg/s at 30 deg of gaze eccentricity, which is lower than what we found in most of our subjects. On the other hand they also reported that the eye drift velocity was 6.8 deg/s at 55 deg. Since the maximal gaze angle reached by all of our subjects was 40 deg, a direct comparison is not possible. Although different from those found in our study, the velocities observed by Gordon and colleagues confirm by themselves a nonlinear growth, which was not further evaluated in their study. Eizenman ([3]) categorized subjects according to whether they developed nystagmus due to fatigue or whether they were showing a sustained nystagmus from the beginning of eccentric fixation. Our results are in line with the values reported by Eizenman at 40 deg for the subjects showing sustained nystagmus.

Although previous studies did not measure eye drift velocities in a continuous range of gaze eccentricities, a decrease of the integrator time constants at large angles of gaze was already reported ([3]). This suggests that the integration process might work differently at different angles. Some models already proposed a nonlinearity in the integration process ([24], [25], [43]). These models, however, dealt with pathological nystagmus (congenital nystagmus, gaze evoked nystagmus and spontaneous nystagmus, respectively)and used eye position nonlinear positive feedback loops to obtain the desired behavior. In a similar way, more recent models proposed the use of dynamic non-linear gains, which depend on eye eccentricities to explain the eye position dependence of rotational vestibulo-ocular reflex behavior ([44], [45], [46], [47]). By appropriately adjusting the eye-eccentricity dependence, all these model structures ([25], [24], [45]) could possibly describe the gaze holding behavior observed in our data. However, our aim was to find a simple function able to encapsulate the main features of the observed dependence of eye drift velocity on eye position, i.e. of the measurable manifestation of the leakiness of the integration process. We showed that for eccentricities between 20 and 40 deg, the linear approximation of the position-velocity curves worsened, while a tangent function can better capture both the weak linear growth of eye drift around primary position and the rapid nonlinear increase observed at larger eccentricities, without the need for two different strategies as suggested before ([25]).

Although the tangent function is not derived from a specific model, the described behavior is consistent with the biological constraints that the brain has to overcome to hold gaze steady ([26], [27]). To illustrate this point we considered neural networks similar to those introduced by Aksay and colleagues ([39]). These authors showed that a network of neurons incorporating some of the known characteristics of those involved in the velocity signal integration in the goldfish can be trained to approximate a perfect integrator within a certain range of eye eccentricities. We simulated a similar network, showing that it is able to mimic the nonlinear behavior that we found experimentally. However, we stress that it is in principle possible to obtain any arbitrary drift pattern by choosing the right parameters and that we are not implying that the tuning strategies we used are actually implemented by the brain. As the goal and the mechanisms used by the brain to tune the network are not known, we did not use our network to fit our data. Instead, our aim was to show that leakiness and non-linearity arise naturally as the behavior of the integrator network in the brain is affected by a number of nonlinearities at neuronal level. Our simulations indeed show that simply considering the inhibitory cutoff, the most known nonlinearity affecting neurons, the network needs to be finely tuned to obtain a linear behavior in the most frequently used eye positions. Inhibitory cutoff is only one of the problems the brain has to overcome to provide integration of an input velocity signal given a limited number of nonlinear neurons. Nonetheless we showed that it may already lead to an outcome which is qualitatively similar to the experimental data. Implementation in the network of nonlinear synaptic activation functions led to simulations that resembled the experimental data more closely, although still quantitatively different, as more complex interactions should be considered to model the actual integration network in the brainstem. In general, whatever the characteristics of the actual integrator network in the brain are, the activity of each neuron should be sustained by the inputs it receives from the others in the network. Moreover such inputs should increase anytime the network needs to reach a new persistent state, required to keep the eye in an increasingly eccentric position. We hypothesize that the nonlinearity we observed occurs due to a progressive saturation of the input that each neuron in the integrating network receives from the others when the eyes approach maximum eccentricity. Given a limited pool of neurons, the brain might optimize the recruitment strategy to obtain the best performance in the range of gaze eccentricity most commonly used. This comes at the cost of saturation of the network output, as well as that of the input to each neuron in the network, beyond a certain angle of gaze eccentricity. This saturation would imply a progressive decline of integration performance, as the needed firing rate cannot be maintained.

## **Conclusion**

We conclude that gaze holding in healthy humans does not follow a linear function, but is much better characterized by a tangent. The nonlinearity of the gaze holding behavior in healthy subjects is well grounded on neuronal physiology and the use of a tangent function provides a compact and simple characterization of healthy behavior to be used as a reference when investigating pathological conditions of gaze holding, e.g. in patients with progressive degenerative vestibulo-cerebellar disease.
